# Probiotics, Maternal Microbiome, and Early-Life Programming: A One Health Perspective on Perinatal and Infant Health

**DOI:** 10.3390/nu18111700

**Published:** 2026-05-27

**Authors:** Valentina Biagioli

**Affiliations:** 1 Microbiota International Clinical Society (MICS), 10153 Turin, Italy; valentina.biagioli@edu.unige.it; 2 Department of Neuroscience, Rehabilitation, Ophthalmology, Genetics, Maternal and Child Health, University of Genoa, 16126 Genoa, Italy

## 1. Introduction

In recent years, the role of the gut microbiota in regulating human health has gained increasing scientific attention, particularly during early life. The perinatal period and early infancy represent a critical window for microbiota establishment and for the metabolic, immunological, and neurodevelopmental programming of the individual. Early microbial colonization is now widely recognized as a key determinant of long-term health, influencing a broad range of physiological and pathological processes [1,2,3].

Within this framework, pregnancy can be conceptualized not merely as a biological phase, but instead as a complex eco-biological window in which maternal physiology integrates environmental exposures, lifestyle factors, and interconnected microbial ecosystems to shape fetal development. From a One Health perspective, the maternal microbiome acts as a dynamic interface between the external environment and the intrauterine milieu, translating ecological signals into immunological, metabolic, and neuroendocrine responses that influence placental function and developmental programming [4].

This Special Issue, “Benefits of Probiotics During Perinatal and Infant Period for Gastrointestinal Health”, brings together six contributions exploring the role of probiotics in microbiota modulation and in promoting gastrointestinal and systemic health during early life. Collectively, these studies provide an integrated overview of current evidence, highlighting both the clinical potential and the remaining gaps in this rapidly evolving field.

## 2. The Microbiota as an Early Determinant of Health

The human microbiota is increasingly recognized as a functional “organ” capable of profoundly influencing host homeostasis through metabolic, immune, and neuroendocrine interactions. During the perinatal period, its composition is highly dynamic and influenced by multiple factors, including mode of delivery, feeding practices, environmental exposures, and maternal health [5].

In this context, the review by [Contribution 1 Mogoş et al.] examines the role of the gut microbiota in early life and the potential of probiotics as a therapeutic strategy for dysbiosis, highlighting their impact on conditions such as allergies and metabolic disorders. Complementarily, [Contribution 2 Biagioli et al.] focuses on the maternal microbiome during pregnancy, emphasizing how microbial imbalances may influence fetal immune, metabolic, and neurodevelopmental trajectories.

Together, these contributions reinforce the concept that early microbiota modulation represents a key determinant of long-term health, supporting a systems-based perspective in which human health is embedded within broader biological and environmental networks.

## 3. From Birth to Weaning: A Critical Window for Microbiota Development

The period from birth to weaning represents a highly dynamic phase for microbiota maturation. Increasing evidence suggests that host–microbe interactions begin in utero and continue through breastfeeding and the introduction of solid foods [1,2,3,4,5,6].

Human milk plays a central role in this process, acting as a source of both microbes and bioactive components that shape neonatal gut colonization and immune development [7,8]. In addition, growing interest in probiotics, prebiotics, and postbiotics reflects efforts to modulate early-life microbiota composition in a targeted manner, promoting the establishment of a balanced and resilient microbial ecosystem.

These findings further support the notion that early nutritional interventions represent powerful tools for influencing long-term health trajectories.

## 4. Probiotic Interventions in the Mother–Infant Dyad

A central focus of this Special Issue is the impact of probiotic supplementation during pregnancy and lactation.

The randomized controlled trial by [Contribution 3 Binda et al.] evaluates the effects of probiotic supplementation in pregnant women and their infants. Supplementation with *Lacticaseibacillus rhamnosus* Rosell^®^-11 and *Bifidobacterium bifidum* HA-132 during the third trimester and lactation reduced infection frequency in mothers and the number of infection days in infants during the first month of life. Although no significant changes were observed in maternal vaginal microbiota, infants exhibited a higher abundance of beneficial gut taxa. The effect was particularly evident in cesarean-delivered infants. These findings support a role for maternal probiotic supplementation in promoting vertical transmission and early microbial colonization. In parallel, the clinical study by [Contribution 4 Pagliarini et al.] shows that maternal supplementation with *Lactobacillus reuteri* during lactation can influence both breast milk composition and neonatal gut microbiota.

These findings highlight the pivotal role of the mother as a mediator of microbial transmission and support the concept that targeted interventions within the mother–infant dyad may promote beneficial microbial colonization.

From a One Health perspective, the maternal microbiome should be viewed as an integrated network spanning gut, vaginal, oral, and mammary niches, collectively regulating systemic inflammation, metabolic homeostasis, and the production of bioactive metabolites, including short-chain fatty acids, bile acids, and tryptophan derivatives [9,10]. Within this interconnected system, vertical transmission of microbes and metabolites represents a key mechanism underlying early-life programming.

## 5. Probiotics in Vulnerable Populations: The Case of Preterm Infants

The use of probiotics in preterm infants represents one of the most studied yet debated areas in neonatal research.

The systematic review and meta-analysis by [Contribution 5 Söderquist Kruth et al.] at the Karolinska Institutet evaluated the effects of probiotic supplementation in extremely preterm infants (<28 weeks of gestation). While probiotics showed a non-significant trend toward improved feeding tolerance and reduced duration of parenteral nutrition, they were associated with a significant reduction in necrotizing enterocolitis (NEC) and all-cause mortality. No consistent effects on growth outcomes were observed. These findings suggest potential clinical benefits of probiotics in high-risk populations, warranting further well-designed trials.

## 6. Translation into Clinical Practice: Knowledge and Barriers

Despite increasing scientific interest, the clinical application of probiotics remains heterogeneous.

The cross-sectional study by [Contribution 6 Rani et al.] reveals considerable variability in pediatric providers’ knowledge and prescribing practices regarding probiotics. This variability reflects both the complexity of the field and the lack of standardized clinical guidelines. More broadly, these findings highlight the challenges of translating microbiome research into practice, particularly in the absence of unified frameworks integrating microbiology, nutrition, clinical medicine, and environmental health-key components of a One Health approach.

## 7. Open Questions and Future Directions

Despite significant progress, several key questions remain unresolved, including identifying the most effective probiotic based on the clinical picture of reference, determining the optimal timing of interventions, and assessing their long-term effects on health.

Advancing the understanding of microbiome-mediated maternal–fetal programming will require integrating mechanistic, translational, and clinical research. Microbiome-targeted interventions during pregnancy, such as dietary modulation, prebiotic, probiotic, and postbiotic supplementation, and lifestyle strategies, have shown promise in influencing maternal microbial composition and metabolite profiles, although randomized evidence remains limited and heterogeneous.

Precision medicine approaches will be essential, given the marked inter-individual variability in microbiota composition, host genetics, and environmental exposures. The integration of multi-omics technologies, including metagenomics, metabolomics, and epigenomics, may enable the identification of individualized microbial and metabolic signatures associated with pregnancy outcomes.

Microbiome-based biomarkers also hold promise for early risk stratification [11]. However, prospective longitudinal studies are needed to validate their predictive value and clinical applicability.

From a public health perspective, early-life microbial exposures influence immune and metabolic programming with implications for chronic conditions such as asthma, obesity, and neurodevelopmental disorders. Integrating microbiome research within a One Health framework emphasizes the importance of combining individual-level interventions with population-level strategies addressing environmental determinants, including urbanization, biodiversity loss, pollution, and industrialized lifestyles.

## 8. Conclusions

This Special Issue provides a comprehensive and updated overview of the role of probiotics in modulating the microbiota during the perinatal and infant periods.

More broadly, pregnancy emerges as a transient yet highly integrated eco-biological system in which maternal physiology, environmental exposures, and microbial ecosystems converge to shape fetal development and early-life health trajectories. The maternal microbiome, spanning multiple niches, functions as a dynamic hub translating ecological signals into metabolic, immunological, and neuroendocrine pathways that influence placental function and developmental programming.

These processes operate across interconnected temporal windows—from preconception through gestation and early postnatal life—highlighting the continuity of maternal influence beyond birth. However, despite significant advances in understanding microbial transmission, metabolite-mediated signaling, and placental integration, translation into routine clinical practice remains limited. Moreover, conceptualizing pregnancy as a modifiable ecological system opens new opportunities for interventions targeting diet, lifestyle, microbial exposures, and environmental determinants within a One Health framework.
